# Challenges in assessing the impact of infection and disease control interventions over the past decade based on the Expanded Special Project for the Elimination of Neglected Topical Diseases (ESPEN) database

**DOI:** 10.1093/trstmh/traf005

**Published:** 2025-02-19

**Authors:** Chi Wai Ng, Rosie Maddren, Roy M Anderson

**Affiliations:** Department of Infections Disease Epidemiology, School of Public Health, Faculty of Medicine, Imperial College London, White City Campus, 90 Wood Lane, London W12 0BZ, UK; Department of Infections Disease Epidemiology, School of Public Health, Faculty of Medicine, Imperial College London, White City Campus, 90 Wood Lane, London W12 0BZ, UK; Department of Infections Disease Epidemiology, School of Public Health, Faculty of Medicine, Imperial College London, White City Campus, 90 Wood Lane, London W12 0BZ, UK

**Keywords:** soil-transmitted helminthiasis (STH), neglected tropical diseases (NTD), epidemiology, control

## Abstract

Over the past 2 decades there has been good progress in the control of many of the neglected tropical diseases (NTDs) treatable by preventative chemotherapy (PC). Continued major drug donations from pharmaceutical companies, support from philanthropic organizations and heightened international recognition of the health impacts of these diseases have each played an important role in lowering the global health burden due to NTDs. However, considerable improvement in data collection is required to accurately assess this progress as we move towards the ‘end game’ of eliminating these infections as a source of morbidity and mortality. The data quality, type and format collected by the Expanded Special Project for the Elimination of Neglected Tropical Diseases database from the African Ministries of Health are discussed and suggestions made for improvements in collection and presentation.

## Origins and purpose of ESPEN

The Expanded Special Project for the Elimination of Neglected Tropical Diseases (ESPEN) was launched in 2016, financially supported by the Bill and Melinda Gates Foundation (BMGF),^1^ with the view to stimulate data collection coordination of neglected tropical disease (NTD) infection levels and mass drug administration (MDA) reporting across the countries enumerated by the World Health Organization (WHO) Regional Office for Africa. Through collaboration with and between the member states and NTD partner organizations, the database portal annually reports programmatic impact, including levels of endemicity (based on the prevalence of infection in defined age groups) and treatment coverage, for the five NTDs eligible for control by preventative chemotherapy (PC-NTDs), i.e. lymphatic filariasis (LF), onchocerciasis, soil-transmitted helminthiasis (STH), schistosomiasis and trachoma.

## Reported progress in control over the period 2013–2022

We examined the records in the ESPEN dataset^2^ over a decade by sampling the endemicity and treatment data from four countries, two from West Africa and two from East Africa: Kenya, Ethiopia, Nigeria and Senegal. By way of illustration, we focus on the data captured for STH. In the ESPEN dataset, endemicity is reported as a category and treatment coverage is defined as the percentage of the target population offered treatment. Such data are provided at the level of health implementation units (IUs), which are administrative divisions used for organizing and implementing health interventions.

**Figure 1.**
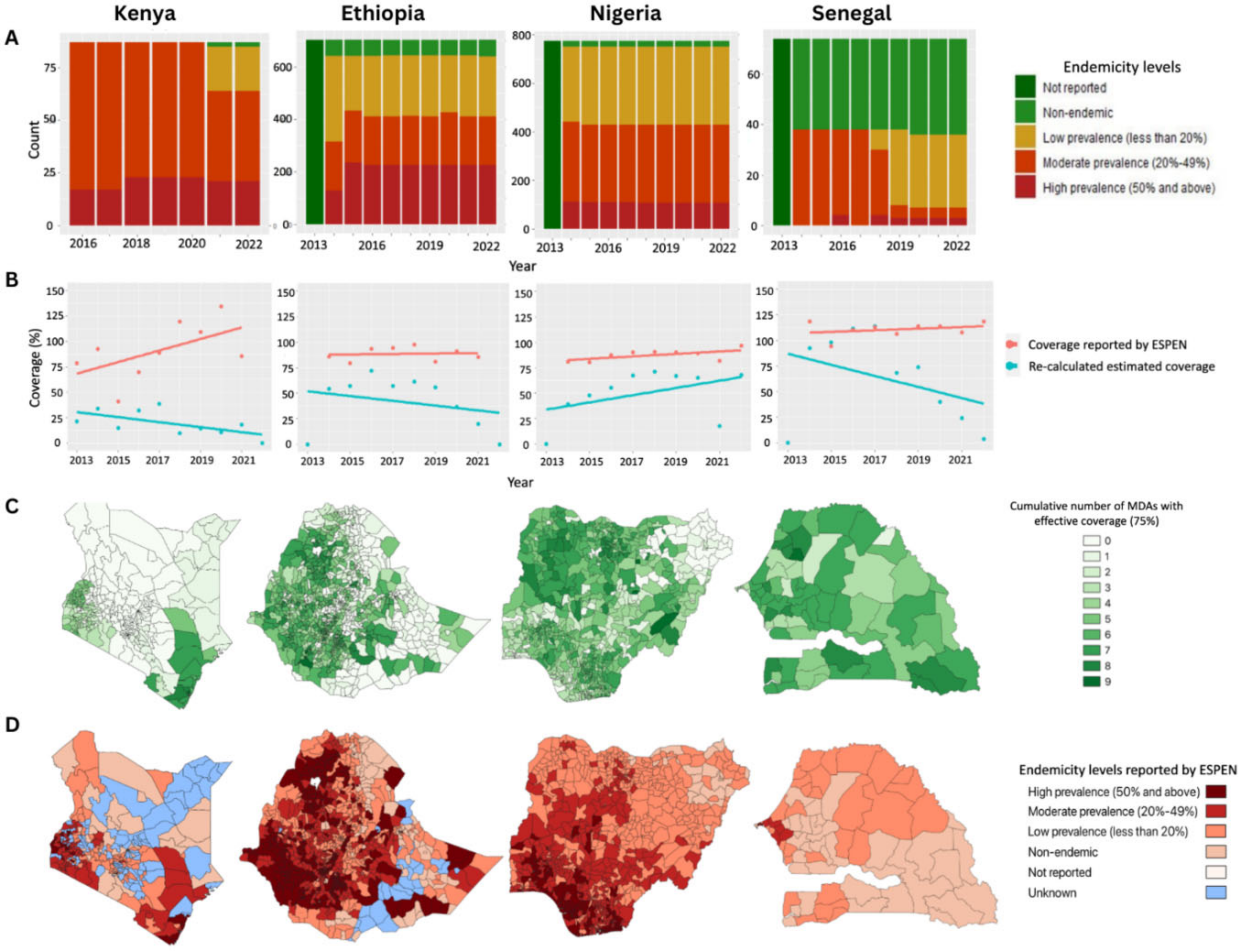
Compilation of ESPEN data regarding reported MDA coverage and endemicity levels of STH in Kenya, Ethiopia, Nigeria and Senegal (from left to right). **(A)** Bar graphs representing the number of health IUs with different endemicity levels from 2013 to 2022. Only IUs with the longest consistent records of endemicity levels throughout the observation period are included. With the exception of Kenya (data records for 2016–2022), the other three countries have records from 2013 to 2022. **(B)** MDA coverage reported by ESPEN compared with that calculated using the US Census Bureau population data from 2013 to 2022. Lines of best fit were added to show the trends in coverage levels. **(C)** Spatial mapping of cumulative effective coverage of IUs from 2013 to 2022. Green represents greater cumulative effective coverage whereas white represents lower coverage. **(D)** Spatial mapping of endemicity level in 2022. Categorical endemicity levels are represented by the colour red. The deeper the red, the higher the endemicity level. Blue represents IUs with no reported data.

As displayed in Figure 1A, the number and proportion of IUs with high levels of infection prevalence generally decreased in the four countries over the period 2013 to 2022. The decrease was most striking in Senegal, where the number of IUs with moderate to high prevalence levels dropped from ≥50% to approximately 10%.

## Problems in data collection

Despite great progress, problems remain in data collection and presentation. The data reported by the dashboard are subject to many inaccuracies, notably contradicting trends between the number of school-age children (SAC) requiring and eligible for treatment and many of the mass drug coverage data points greater than 100%. This raises questions surrounding their validity and value as a measure of control impact. These inaccuracies can be broadly labelled as errors stemming from both the numerator and the denominator, plus the nature of the epidemiological data recorded and the spatial scale at which data are presented.^3^

These challenges can be examined across a series of key dimensions in data quality.

### Accuracy and timeliness challenges

The denominator for the calculation of MDA coverage should be the total count of SAC in endemic IUs. As shown in Figure 1A, the endemicity in the sampled countries remained relatively stable, therefore the number of IUs requiring MDA would have been unchanged. The number of SAC eligible for treatment would be expected to increase in line with population growth estimates. This is not seen in the data.

The demographic data used are rarely accurate. This is a common problem in countries where population census data collection is infrequent and birth rates plus people movements are high. To obtain a denominator, the WHO applies the United Nations population projections, which model age groups stratified into 5-y intervals, on a 5-y basis from the last conducted census. Alternatively, other sources of data exist, such as projections made by the US Census Bureau, which provides annual estimates of the number of SAC for all countries in the world.

To assess the reliability of the ESPEN coverage data, we applied estimates of SAC (ages 5–14 y), published by US Census Bureau as an alternative denominator. As shown in Figure 1B, this recalculated estimated coverage is consistently less than that reported by ESPEN for each of the four country scenarios. The difference between the coverage figures calculated by ESPEN, or through this enhanced denominator, range from approximately 6% to 120%. The mismatch highlights a real uncertainty surrounding which denominator to use in countries where annual growth rates lie between 2% and 3%.

### Completeness and uniqueness

The numerator used to calculate treatment coverage does not capture all the national control efforts. It is limited to that reported by federal ministries of health (FMoHs). In Nigeria, for example, the Federal Ministry of Education also conducts MDA in schools, yet only the FMoH is affiliated with the WHO and therefore only data from their sanctioned programs are submitted to ESPEN. In addition, many deworming programs conducted using financial support from philanthropic organizations are not always captured by FMoHs.

The potential for duplicate reporting exists where multiple programs operate in the same areas. The current system struggles to distinguish unique treatments when various organizations conduct MDAs in overlapping regions.

### Data integrity and validity

Underreporting of treatments combined with uncertainty in the accuracy of the denominator employed reduces confidence in the reported coverage data, which can in some cases be >100%, as seen in Kenya and Senegal (see Figure 1B). In Senegal, in all the coverage data reported from 2013 to 2022 in ESPEN, 59% of the data points are >100%.

Finally, ESPEN only publishes categorical endemicity rather than the estimated prevalence of infection levels for IU-level data. High prevalence is defined as >50%, moderate as 20–50% and low as <20%. It is not always clear which age groups are included in the specification of endemicity, how it was calculated, what sample size was employed or when the estimates were made. While the purpose of the ESPEN dataset is to advise governments on MDA implementation and NTD control progress in IUs, the absence of quantitative values and information on how measurements are made makes it challenging to assess the accuracy of assigned endemicity levels, thus hindering further analysis and interpretation. As many subregions are now reported to be reaching low to moderate levels of STH prevalence, accessing fine-scale spatial data is of increasing importance in identifying the remaining hot spots of infection and targeting control activity to them. Making available within the ESPEN database the data used to inform the categorization of endemicity and the age groups sampled would greatly improve assessment of progress in control.

### MDA coverage and infection levels

Areas with high cumulative effective MDA coverage over the period 2013 to 2022, as plotted in Figure 1C, have a strong positive correlation with the endemicity levels in 2022 (Figure 1D). This, at first sight, seems very surprising given that prevalence in 2022 is expected to be lowest in the units that have had the most MDA rounds over the previous 10 y. Explanation of this counterintuitive pattern may lie in the fact that the high treatment coverage sites are settings with the highest transmission potentials (high basic reproductive numbers, R_0_), which necessitate high and frequent MDA coverage to lower infection levels, and, as a consequence, they have been the target of more rounds of chemotherapy than the average health unit. Linked to this possible explanation is the commonly observed phenomena of the predisposition of individuals to repeated high levels of reinfection after each round of MDA.^4^ However, it may also result from inaccuracies in data collection, either of coverage, infection levels or both.

It is also worth noting that MDAs were restricted to SAC. To date, pre-SAC data are not available in ESPEN. The incorporation of multiple age groups is crucial for tracking endemicity levels across the whole population. This is especially important for areas where hookworm is the dominant STH, since for this parasite most infection is present in the adult age classes. In 2022, the WHO released the fourth version of its recommended STH treatment strategies, which includes pre-SAC and women of reproductive age.^5^ Hopefully, this will result in data collection broadening to include age groups other than SAC.

## How to improve ESPEN data quality?

Clear specifications by countries of what data sources were used to calculate coverage and endemicity levels reported in the portal would improve assessment of progress in NTD control. The application of appropriate and recent denominators to all calculations would greatly facilitate countries to better evaluate their programmatic impact. Other publicly available datasets, such as those presented by the US Census Bureau, or alternatives that provide annually renewed data at continuous age-range intervals would be preferrable. Additionally, ensuring the data reported to the ESPEN portal are representative of the whole national program, and not just that actioned by the ministries of health, would improve our understanding of the national response to control infections. These improvements to data reporting will depend in part on strengthening government–philanthropic organization partnerships to support better data collection.

While these improvements would enhance ESPEN's utility, it is important to recognize the inherent limitations of such a centralized database system. ESPEN alone cannot provide the granular epidemiological insights needed for precise disease control. Countries should develop complementary epidemiological tools, particularly as prevalence levels decline. These could include cross-sectional surveys with stratified sampling designs, local surveillance systems and detailed mapping of transmission hot spots. Such tools, when used alongside ESPEN, would provide more reliable coverage data and better characterize the spatial distribution of infections. The combination of ESPEN's broad overview with locally tailored monitoring systems would create a more comprehensive approach to NTD control.

Finally, the stratification of the data provided online is not conducive to fine-scale monitoring. Chiefly, the portal was created as a broad-stroke guide to monitor progress toward control of the five chosen PC-NTDs. However, as an increasing number of countries contain subregions of very low prevalence, fine-scale monitoring to identify hot spots of continued transmission and areas for improved MDA coverage is increasingly needed. Therefore, to simply remove the stratification of endemicity into categories and report the continuous data that informed such calculations would be a vast improvement in providing insights into the impact of control.

## Conclusions

The ESPEN dataset has been influential since its introduction in 2016. The data reported has been used to help validate the elimination of both trachoma in Ghana and LF in Togo and Malawi. The database has been the result of much work by member states and NTD partners, and managing, standardizing and analysing large datasets is never easy across ministries with varied resources and capabilities. However, as the WHO targets focus increasingly on fine-scale monitoring and evaluation, there is scope for much improvement in both the data collected and its quality. The structure and management of ESPEN data quality relating to the numerator, the denominator and the spatial scale at which it is reported are central to accurate assessment of control progress. As infection levels decrease due to high levels of MDA delivery over the past 2 decades, more resources are required to monitor how best to reach and implement the endgame for these infections. Finally, it should be noted that the five PC-NTDs do not observe geopolitical borders. Therefore, it would be beneficial for AFRO to collaborate with the Pan American Health Organization, WHO South-East Asia (SEARO) and WHO Western Pacific to develop a high-quality truly global database.

## Data Availability

The data underlying this article are available from ESPEN (https://espen.afro.who.int/) and the US Census Bureau (https://www.census.gov/). The datasets were derived from sources in the public domain and ESPEN portal (https://www.census.gov/programs-surveys/international-programs/about/idb.html and https://espen.afro.who.int/tools-resources/data-reporting-tools/espen-portal-joint-application-package-jap-upload-tool).
